# Crude extract of Jatobá leaves promotes canine osteosarcoma cell D17 proliferation

**DOI:** 10.14202/vetworld.2022.1283-1289

**Published:** 2022-05-24

**Authors:** V. S. Vieira, V. S. Cruz, L. L. Nepomuceno, N. P. Soares, E. Arnhold, W. F. P. Teixeira, D. S. Vieira, J. C. A. Borges, F. M. Paixão, E. G. Araújo

**Affiliations:** 1 Multi-User Laboratory for the Evaluation of Molecules Cells and Tissues, Veterinary and Zootechnical School, Federal University of Goiás, Campus Samambaia, Avenida Esperança, Goiânia, GO, 74690-900, Brazil; 2 Department of Veterinary Medicine, Catholic University Center of East Minas, Campus Coronel Fabriciano, Av. Pres. Tancredo de Almeida Neves, 3500, B - Morada do Vale B, Cel. Fabriciano MG, 35170-056, Brazil; 3 Embrapa Southeast Livestock, São Carlos, São Paulo, Brazil, Rod, Washington Luiz, Km 234 - Fazenda Canchim, São Carlos - SP, 13560-970, Brazil

**Keywords:** antineoplastic chemotherapy, biodiversity, bone tumors, *Hymenaea martiana* Hayne, medicinal plants

## Abstract

**Background and Aim:**

New substances for neoplasm treatment have to be carefully studied to minimize adverse effects and prevent disease progression stimulation. Jatobá is a typical tree of the *Cerrado* and *Caatinga* biome, with antifungal, antimicrobial, larvicide, antioxidant, and antiproliferative properties. This study aimed to investigate the action of the crude extract of Jatobá leaves (EBFJ) on canine osteosarcoma (CO) cells and analyze the expression of biomarkers in neoplasm progression.

**Materials and Methods:**

D17 cells were cultured and subjected to treatment with EBFJ at different concentrations (10 μg/mL; 100 μg/mL; 1000 μg/mL; 2000 μg/mL; and 5000 μg/mL) and exposure times (24 h, 48 h, and 72 h). The tetrazolium reduction assay and the immunocytochemistry technique, with anti-Bcl2, anti-p53, and anti-Ki-67 antibodies, were used to observe the effect of the extract on cell proliferation.

**Results:**

Doses of 2000 µg and 5000 µg had cell viability of 300.80% and 361.84%, respectively. The extract did not show significant cytotoxicity of samples with the control group. The confluence of cells, the number of labeled cells, and the expression of Bcl2, Ki-67, and p53 were higher in the groups treated with EBFJ, with a statistical difference from the group without treatment.

**Conclusion:**

EBFJ was not cytotoxic and had a proliferative effect on CO D17 cells. The confluence of cells, the number of labeled cells, and the expression of Bcl2, Ki-67, and p53 were higher in the groups treated with the extract.

## Introduction

Canine osteosarcoma (CO) is the most common primary bone neoplasm in dogs [1-3]. The tumor is aggressive due to high cell proliferation and rapid metastasis development. The prognosis for CO remains reserved despite scientific advances related to different types of therapy [3] since <20% of dogs survive for >2 years after diagnosis [4]. The unknown role involved in CO genesis and progression indicates the need for elucidation, as well as the use of biomarkers to assist in early diagnosis, predict clinical outcomes, and monitor the treatment effectiveness [5].

Another issue is the search for new drugs to innovate strategies to prevent CO progression. Brazil has five biogeographic provinces, including the Cerrado biome, with biodiversity that allows both consumptions through popular knowledge and the development of new drugs by the pharmaceutical industry [6]. Highlighting that each investigated substance has a selection of molecules with specific mechanisms for each type of neoplasia is important [7]. Therefore, the use of new substances, as well as therapies without scientific evidence for a given neoplasm, needs to be carefully reviewed to minimize adverse effects and contraindications and even prevent disease progression stimulation.

Folk medicine is passed down through generations, which usually consume parts of plants, such as roots, leaves, fruits, or seeds, that are easily obtained in everyday life based on some known effect on health promotion, disease treatment, and symptom improvement [8]. Various plants are also used in pets to replace antibiotics, treat heart, digestive, respiratory, liver, and urinary problems, seasonal allergies, atopic dermatitis, endoparasites, and viral infections, improve reproductive life, and improve the quality of life of patients with cancer [9].

As medicinal herbs originate from nature, users often believe that they are benefiting from not using pharmaceutical drugs and that problems are unlikely to occur, even in the long term. However, the material and processes used to obtain them, as well as their misuse, without information about care and contraindications together with an inadequate process for notifying adverse effects, can pose several risk factors [8]. An example of this situation was observed in university hospitals in Korea, where an incidence of 23.9% of adverse events associated with the use of folk medicine was reported by Yoo *et al*. [10].

Known as Jatobá or Jatobá-do-mato, *Hymenaea martiana* Hayne is a typical tree of the *Cerrado* and *Caatinga* biomes. Plants of the genus are used as medicinal plants for inflammatory process and bacterial infection treatment [11]. The popular use of *Hymenaea* spp. is made through infusions of the bark, fruit, and leaves as a tonic, expectorant, hepatoprotective, and vermifuge [12]. Some properties were substantiated by pharmacological studies, such as antifungal and antimicrobial [13], larvicidal [14], antioxidant [15,16], and antiproliferative activities in some cancer cells [14,17,18].

This study aimed to investigate the activity of the crude ethanol extract of *H. martiana* Hayne leaves on CO cells and analyze the biomarker expression in neoplasm progression.

## Materials and Methods

### Ethical approval

Ethical approval is not required for this type of study as it does not include live animals. Cells used in the experiment are from a culture commission acquired directly from the cell bank.

### Study period and location

The study was conducted from January 2019 to July 2019. The experiment was conducted at the Multi-user Laboratory for the Evaluation of Molecules, Cells and Tissues, School of Veterinary and Animal Sciences, Federal University of Goiás.

### Plant material and extract preparation

Leaves were collected from a specimen deposited in the Vale do São Francisco Herbarium, identified as plant exsiccates 21868. The production of the crude ethanol extract of the *H. martiana* Hayne leaf was adapted from the method by Peixoto *et al*. [19]. After collecting the leaves, they remained in an air circulating oven at 45°C for 7 days before maceration.

The extract was analyzed using two methods, including high-performance liquid chromatography (HPLC) and HPLC with diode array detection (HPLC-DAD). In the HPLC analysis, *H. martiana* Hayne leaves were subjected to solid-phase extraction (SPE C-18). The cartridge was previously activated using methanol at 10 mL and ultrapure water at 10 mL, and then, 100 mg extract was solubilized in 500 μL acidified water (pH=2 with HCl) and 500 μL MeOH. After loading the extract into the cartridge, 10 mL of ultrapure water was added, and soon after, the fraction with organic compounds was eluted with 10 mL of methanol. This methanol fraction was analyzed by HPLC.

HPLC-DAD analysis was performed using a Shimadzu Prominence LC-20AT high-efficiency liquid chromatography with a diode array detector (SPDM20), SIL-20AC automatic injector, CTO-20A oven, and DGU-20A5 degasser. Chromatographic separation was performed with a Luna C-18 column (250 mm×4.6 mm×5 μm, Phenomenex). A mixture of H_2_O: formic acid (99:1, Solvent A) and methanol (Solvent B) was used as the mobile phase, with the elution system of 0-10 min, 90-100% B, 10-30 min, 100% B, the flow of 1.0 mL/min, the temperature of 35°C, and the wavelength of 320 nm, which were used for monitoring. Nylon (Whatman) filters at 0.45 μm were used for sample filtration. All solvents used were HPLC grade. Samples were refrigerated at 4°C and diluted in dimethyl sulfoxide (DMSO, Cultilab, Campinas, Brazil), at the concentrations designed for this experiment.

### Cell cultivation

Canine metastatic osteogenic osteosarcoma cells (D-17, BCRJ 0276, Lot 000573, Passage 239, *Canis familiaris*), originating from the American Type Culture Collection (Manassas, VA, USA), were purchased from the Rio de Janeiro Cell Bank (UFRJ – Rio de Janeiro, Brazil). Cells were grown in Dulbecco’s modified Eagle culture medium (DMEM) supplemented with 10% fetal bovine serum, penicillin, and streptomycin (10,000 IU/mL, 10 mg/mL), and amphotericin B and glutamine L (all reagents from Cultilab, Campinas, Brazil) and kept in a humidified incubator at 37°C with an atmosphere of 5% CO_2_.

### Cell viability and cytotoxicity assay

At the end of the cultivation process, cells were counted in a Neubauer chamber and seeded in 96-well plates, with 200 μL DMEM medium, at a concentration of 1×10^4^ per well. Plates were kept in a humidified incubator at 37°C and in a 5% CO_2_ atmosphere for 24 h. Afterward, the medium was discarded and the wells were treated or not with extract of Jatobá leaves (EBFJ) diluted in DMSO, at concentrations of 10 μg/mL, 100 μg/mL, 1000 μg/mL, 2000 μg/mL, and 5000 μg/mL, with an exposure time of 24, 48, and 72 h. The negative control group (CG) was treated with only DMSO. The wells were randomly treated, and the technique was performed in quintuplicate, with three independent experiments.

After the treatment period (24, 48, and 72 h), the tetrazolium salt reduction 3-(4,5-dimethyl-2-thiazolyl) -2,5-diphenyl-2H-tetrazolium (MTT) assay was used, by the pyruvate dehydrogenase enzymatic complex, presents in the matrix of mitochondria. The medium was discarded and 10 μL of tetrazolium MTT was added to each well. Plates were incubated for 3 h. To quench the reaction, 50 μL of 10% sodium dodecyl sulfate (SDS – Vivantis Biochemical) diluted in HCl (0.01N) was added per well, and the plates remained incubated for 24 h at room temperature (26ºC). The optical density was quantified in a spectrophotometer (ELX 800 Universal Microplate Reader, Federal University of Goiás, Brazil).

Cell viability was determined by the equation:

Treatment abs/control abs) × 100

Where, CV is cell viability and abs is absorbance.

Cytotoxicity was determined by the equation:

100 − [(treatment abs/control abs) ×100]

Where, CT is cytotoxicity and abs is absorbance.

The data used came from three independent studies. Comparisons were conducted using the analysis of variance and the Scott-Knott test, with a 5% significance level, R software [20], and R easyanova package [21].

### Immunocytochemistry

D-17 cells were seeded on Falcon™ chamber slides (BD Falcon Culture Slides, Federal University of Goiás), at a concentration of 1×10^4^, cultured, and exposed to EBFJ for 48 h at concentrations with a statistically significant difference in the cell viability and cytotoxicity assay (0 μg/mL, 1000 μg/mL, 2000 μg/mL, and 5000 μg/mL). Afterward, cells were fixed with 4% paraformaldehyde for 30 min.

All steps of the technique were performed by the Bond-Max Fully Automated IHC-ISH (immunocytochemistry) automatic processor (Leica Biosystems, Federal University of Goiás), with the following reagents: Bond Polymer Refine Detection (Federal University of Goiás), Bond™ Wash Solution, Bond™ Epitope Retrieval 1, Bond Dewax Solution, Bond™ Epitope Retrieval 2, Bond 3,3’-diaminobenzidine chromogen, and Bond hematoxylin. Antibodies used were anti-Bcl2 (SC783 rabbit polyclonal, Santa Cruz Biotechnology, Brazil), anti-p53 (SC71785 mouse monoclonal, Santa Cruz Biotechnology), and anti-Ki-67 (SC15402 rabbit polyclonal, Santa Cruz Biotechnology), at 1:500 dilutions in 1.5% bovine serum albumin. A group free of the primary antibody was used as a control for the reaction.

The total cell count was performed in five different fields and the percentage of positive cells, which was marked by the antibody, adapted from Fedchenko and Reifenrath [22] to assess immunostaining. Cells in shades of brown, partially or completely, were considered positive; whereas, cells without labeling, only in shades of blue due to counterstaining with hematoxylin, were considered negative. The positive stained cell ratio (RCCP) was calculated by the ratio of the number of stained cells and the total number of cells multiplied by 100. With this value, categories were assigned as follows: 0 for 0%; 1 for <1%; 2 for 1-10%, 3 for 11-33%, 4 for 34-66%, and 5 for >67%.

A blind semi-quantitative analysis was performed, using scores from 0 to 3 according to the shades of a colorimetric scale for the expression intensity score (EIS). Then, the semi-quantitative combined scoring system (SPCS), Allred [22] score, was adopted, which was defined by the sum of the RCCP with the EIS, after calculating the mean, standard deviation, and exclusion of outliers. Thus, the SPCS scale comprised values from 0 to 8.

### Statistical analysis

Statistical analysis was run using analysis of variance and the Kruskal–Wallis test, p<0.05, R software [20], and R easyanova package [21].

## Results

### Cell viability and cytotoxicity assay

**Figure-1:**
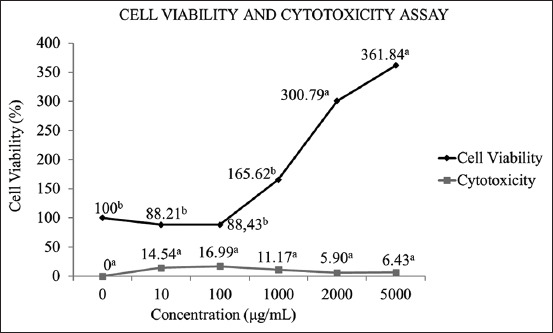
Viability and cytotoxicity assay by tetrazolium salt reduction in canine osteosarcoma cells exposed to a crude extract of Jatobá leaves. Lowercase letters indicate significant differences, Scott-Knott test, p<0.05.

The experiment was conducted with dose and exposure time to determine whether EBFJ could modify the viability of D17 cells. Data used were obtained from three independent studies. Cells were treated with increasing concentrations, which were not affected by the hour or hour/treatment interaction; thus, no difference was observed between the exposure time; that is, the action of the extract was the same in 24, 48, and 72 h. The treatment means were compared, considering all hours together, and observing the extract with a significant proliferative effect was possible, especially at the highest concentrations, where the doses of 2000 µg and 5000 µg had cell viability of 300.80% and 361.84%, respectively, as shown in Figure-1. The negative cytotoxicity values were replaced with 0 and, simultaneously, the extract did not show significant cytotoxicity of the samples with the CG (Figure-1).

### Immunocytochemistry

**Figure-2:**
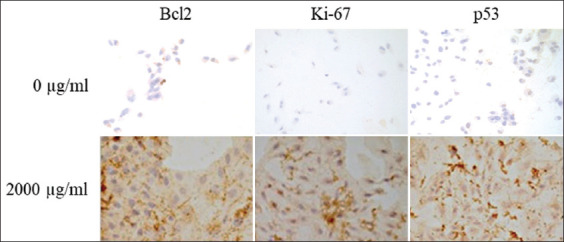
Photomicrographs of slides containing D-17 canine osteosarcoma cells, exposed to Jatobá leaf extract at a concentration of 2000 µg/mL for 48 h, subjected to immunocytochemical assay by Bond-Max automated processor (Leica). Anti-Bcl2, anti-Ki-67, and anti-p53 antibodies. 3,3’-diaminobenzidine, hematoxylin counterstain. 62 µm scale. Fields with positive stained cell ratio (RCCP) 3, EIE 1 and semi-quantitative combined scoring system (SPCS) 4, at 0 µg/mL. Fields with RCCP 5, EIE 3, and SPCS 8, at 2000 µg/mL.

D-17 cells showed Bcl2, Ki-67, and p53 labeling in the nucleus, cytoplasm, and membrane in all groups. The confluence of cells, the number of labeled cells, and the expression of proteins were higher in the groups treated with EBFJ (Figure-2).

No statistical difference was found in the ratio of cells that are positively stained by Bcl2 and Ki-67 between the analyzed groups. The difference was found in the ratio of positive cells stained by p53 and was greater in groups treated with EBFG (1000 µg/mL, 2000 µg/mL, and 5000 µg/mL) than in the non-treated group (0 µg/mL). A significant difference was found in the Bcl2, Ki-67, and p53 EIS, which was lower in the non-treated groups and increased with the rise in EBFJ concentration. The semi-quantitative combined scoring system showed a statistical difference between the groups treated with EBFJ, with the group without treatment, in the three antibodies analyzed. The increase in SPCS of Bcl2 and Ki-67 was dose-dependent (Table-1).

**Table 1:** Evaluation of immunostaining in D-17 cells of canine osteosarcoma, exposed to crude EBFJ. Mean values were observed in the RCCP, the EIS, and the SPCS.

Antibody	EBFJ	RCCP (0-5)	EIS (0-3)	SPCS (0-8)
Bcl2	0 μg/mL	4.57^A^	1.43^A^	6.00^A^
	1000 μg/mL	5.00^A^	2.00^B^	7.00^B^
	2000 μg/mL	5.00^A^	2.00^B^	7.00^B^
	5000 μg/mL	5.00^A^	2.80^C^	7.80^C^
Ki-67	0 μg/mL	5.00^A^	1.00^A^	6.00^A^
	1000 μg/mL	5.00^A^	2.00^B^	7.00^B^
	2000 μg/mL	4.75^A^	2.25^B^	7.00^B^
	5000 μg/mL	5.00^A^	3.00^C^	8.00^C^
p53	0 μg/mL	3.40^A^	1.00^A^	4.40^A^
	1000 μg/mL	5.00^B^	2.00^B^	7.00^B^
	2000 μg/mL	5.00^B^	2.40^C^	7.40^B^
	5000 μg/mL	5.00^B^	2.00^B^	7.00^B^

Different uppercase letters, in the same row, indicate significant difference by antibody, P*<*0.05, Kruskal– Wallis. EBFJ=Extract of Jatobá leaves, RCCP=Positive stained cell ratio, EIS=Expression intensity score, SPCS=Semi-quantitative combined scoring system

## Discussion

This study was conducted using a crude extract of *H. martiana* Hayne leaves. This information is relevant since the action of leaves of this species or even of this genus, on CO cells, was scarcely studied, as well as its activity on animal cells.

The viability results of the tetrazolium salt reduction assay in CO cells that are exposed to the crude EBFJ demonstrated that EBFJ significantly increased the viability of CO cells. A slight inhibitory effect was found at the lowest concentrations but without significant difference from the CG. Differently, the hydroethanolic extract of *Hymenaea courbaril* seeds, in murine melanoma cells (B16F10-Nex2), resulted in 50% cell viability inhibition at a concentration of 50 μg/mL [23].

Similar to this study, Jayaprakasam *et al*. [24] observed increased cell viability in human breast cancer (MCF-7), glioblastoma (SF268), colon cancer (HCT-116), and gastric adenocarcinoma tumor lines (AGS), which are exposed to secondary metabolites isolated from the fruit of *H. courbaril*. Studies with xyloglucan polysaccharides, from the fruit of *H. courbaril*, in cervical cancer cells (HeLa), revealed increased cell viability, suggesting an inducing effect on cell proliferation [25].

Therefore, considering the idea of cell proliferation induction in light of the results obtained in this experiment is possible, especially at concentrations of 2000 µg and 5000 µg, which showed cell viability of 300.80% and 361.84%, respectively. This effect is probably related to the increased proliferative capacity of CO cells compared to the CG, without exposure to the extract. Some plant secondary metabolites are assumed to act on deoxyribonucleic acid (DNA) through covalent bonding or intercalation; thus, cell proliferation is likely to occur if the alkylated bases are not properly repaired or the cell is not directed to apoptosis [26-29].

Notably, protective activities and antioxidant properties are described in the genus *Hymenaea* under different oxidative stress conditions [30]. However, the promotion of proliferation is undesirable in neoplastic cells, especially in CO, notorious for its systemic evolution, due to the high cell propagation and the rapid metastasis. EBFJ showed no cytotoxicity in CO cells under the conditions of this experiment. The extract exhibited minimum cytotoxicity values, without significant difference from the CG. Conversely, the extract from *H. martiana* leaves promoted cytotoxic activity on canine mammary adenocarcinoma cells (AF-720) at a dosage of 25 mg/mL [31].

Possibly, differences among species and the parts of the plant used in the composition of the extract interfere with the cytotoxic activity in different neoplastic cell types. Therefore, the choice of leaves to obtain the extract for this experiment was influenced by the knowledge that the popular use of *Hymenaea* spp. is made through infusions of the bark, fruit, and leaves^5R^. Conventionally, the use of medicinal plants is considered safe, but some reports have shown that the continuous use of these plants can be associated with several injuries [32,33].

We investigated the mechanism by which EBFJ can increase cell viability through immunocytochemistry. Bcl2 expression increased with rising EBFJ concentrations. The increased Bcl2 expression in cell lines indicates cell proliferation promotion; however, this protein can prevent programmed cell death and allow neoplastic cell survival, unlike other oncogenic proteins [34]. The anti-apoptotic function revealed by the overexpression of Bcl2 may be related to the aggressiveness of the neoplasm, providing resistance to therapeutic modalities, thus indicating an unfavorable prognosis [35]. Therefore, the observed increase in Bcl2 expression is likely related to the ability of EBFJ to stimulate anti-apoptotic activity and allow D17 CO cell survival.

A significant difference was found in the Ki-67 EIS, a marker of cell proliferation present in the G1, S, and G2 phases of the cell cycle [36]. The increased Ki-67 index is correlated with tumor progression and metastasis [37]. In non-Hodgkin lymphoma and large bowel, cervical, and uterine cancer, a correlation between Ki-67 expression and a shorter patient survival time was found [37-39]. Thus, possibly, EBFJ provided the proliferation of D17 CO cells.

Herein, the ratio of positive staining of mutant p53 was higher in the groups treated with EBFG, with a significant difference in the EIS of that protein, according to the increased EBFG concentration. The opposite result was found in D17 cells treated with beta-lapachone [2], curcumin [40], and the ethanol extract of pequi peel [41], where the expression of mutant p53 decreased with increasing dosages and exposure time.

Mutations in the TP53 gene occur in the DNA-binding domain, which resulted in a loss of tumor suppressor function and new gain-of-function mutation promotion, which prevents neoplastic cell death, increases conventional treatment resistance, and boosts tumorigenesis, such as an oncogenic protein [42,43]. Such mutations are variable between cancer types due to several carcinogenic agents and can occur at different cancer stages. Mutation at advanced tumor stages plays a key role in tumor proliferation, aggressiveness, and invasion [44]. Supposedly, EBFJ may have performed a function similar to that of a carcinogenic agent, stimulating the mutation of p53 and promoting the proliferation of D17 cells even in the already constituted neoplasm stages.

Our results reinforce the concern with the easy availability of the culture of popular use, which is directly related to self-medication and the administration of plant parts and their derivatives to pets, without professional prescription, even in developed countries. This common practice can be inappropriately used as an alternative in dogs with osteosarcoma, seeking relief for symptoms, such as intermittent pain caused by bone lysis resulting from neoplastic development, or as an attempt to avoid amputation of the involved limb, as the surgical treatment of choice [45]. However, plants can have subacute and chronic toxicity, which are difficult to detect by traditional use or by clinical research studies [46], in addition to substances that can cause serious disturbances, including carcinogenesis [47].

## Conclusion

EBFG may be non-cytotoxic and had a proliferative effect on D17 CO cells, especially at higher concentrations of 2000 µg and 5000 µg. The confluence of cells, the number of labeled cells, and the expression of Bcl2, Ki-67, and p53 were higher in groups that were treated with EBFG.

## Authors’ Contributions

VSV, VSC, LLN, and NPS: Conception and design of the study, acquisition, analysis and interpretation of data, drafted and critically revised the manuscript. JCAB, WFPT, DSV, and FMP: Contributed to conception, analysis of data, participated in design of the study, and interpretation of data. EA and EGA: Supported to analysis and critically revised the manuscript. All authors read and approved the final manuscript.
